# The Full Value of Vaccine Assessments (FVVA): a framework for assessing and communicating the value of vaccines for investment and introduction decision-making

**DOI:** 10.1186/s12916-023-02929-0

**Published:** 2023-07-04

**Authors:** Raymond Hutubessy, Jeremy A. Lauer, Birgitte Giersing, So Yoon Sim, Mark Jit, David Kaslow, Siobhan Botwright

**Affiliations:** 1 Immunization, Vaccines and Biologicals Department, World Health Organization, 20 Avenue Appia, CH-1211 Geneva, Switzerland; 2 Strathclyde Business School, University of Strathclyde, Glasgow, UK; 3 Department of Infectious Disease Epidemiology, London School of Hygiene and Tropical Medicine, London, UK; 4 PATH Center for Vaccine Innovation and Access, Seattle, USA

**Keywords:** Vaccines, Immunization, Research and development, Public health, Economic evaluations, Health technology assessment

## Abstract

**Background:**

Several economic obstacles can deter the development and use of vaccines. This can lead to limited product options for some diseases, delays in new product development, and inequitable access to vaccines. Although seemingly distinct, these obstacles are actually interrelated and therefore need to be addressed through a single over-arching strategy encompassing all stakeholders.

**Methods:**

To help overcome these obstacles, we propose a new approach, the Full Value of Vaccines Assessments (FVVA) framework, to guide the assessment and communication of the value of a vaccine. The FVVA framework is designed to facilitate alignment across key stakeholders and to enhance decision-making around investment in vaccine development, policy-making, procurement, and introduction, particularly for vaccines intended for use in low- and middle-income countries.

**Results:**

The FVVA framework has three key elements. First, to enhance *assessment*, existing value-assessment methods and tools are adapted to include broader benefits of vaccines as well as opportunity costs borne by stakeholders. Second, to improve *decision-making*, a deliberative process is required to recognize the agency of stakeholders and to ensure country ownership of decision-making and priority setting. Third, the FVVA framework provides a consistent and evidence-based approach that facilitates *communication* about the full value of vaccines, helping to enhance alignment and coordination across diverse stakeholders.

**Conclusions:**

The FVVA framework provides guidance for stakeholders organizing global-level efforts to promote investment in vaccines that are priorities for LMICs. By providing a more holistic view of the benefits of vaccines, its application also has the potential to encourage greater take-up by countries, thereby leading to more sustainable and equitable impacts of vaccines and immunization programmes.

**Supplementary Information:**

The online version contains supplementary material available at 10.1186/s12916-023-02929-0.

## Highlights

1. What is already known on this subject?

While existing ‘pull’ and ‘push’ mechanisms to incentivize new vaccine development can enhance coordination across multiple stakeholders with different agendas, these mechanisms have not fully addressed interrelated obstacles for the optimal development, use, and impact of vaccines and immunization programmes in an integrated and holistic way.

2. What this study adds?

This paper presents a conceptual framework, the Full Value of Vaccine Assessments (FVVA), which outlines coherent, organizing principles to guide the assessment of the value of vaccines, promote communication across stakeholders, and facilitate more informed country ownership of decision-making. Application of the FVVA approach has the potential to expand the sustainable and equitable impact of vaccines and immunization programmes.

3. Policy implications

The FVVA approach outlined here will inform global-level efforts to enhance coordination among diverse stakeholders involved in policy- and decision-making related to the development and implementation of new vaccines.

**Supplementary Information:**

The online version contains supplementary material available at 10.1186/s12916-023-02929-0.

## Background

The optimal development, use, and impact of vaccines are hindered by a number of interrelated obstacles. While most of these obstacles are not new, many of them have been brought to the forefront of public debates around the urgent need to develop and deploy a safe, effective and affordable vaccine, particularly in low- and middle-income countries (LMICs).

**Fig. 1:**
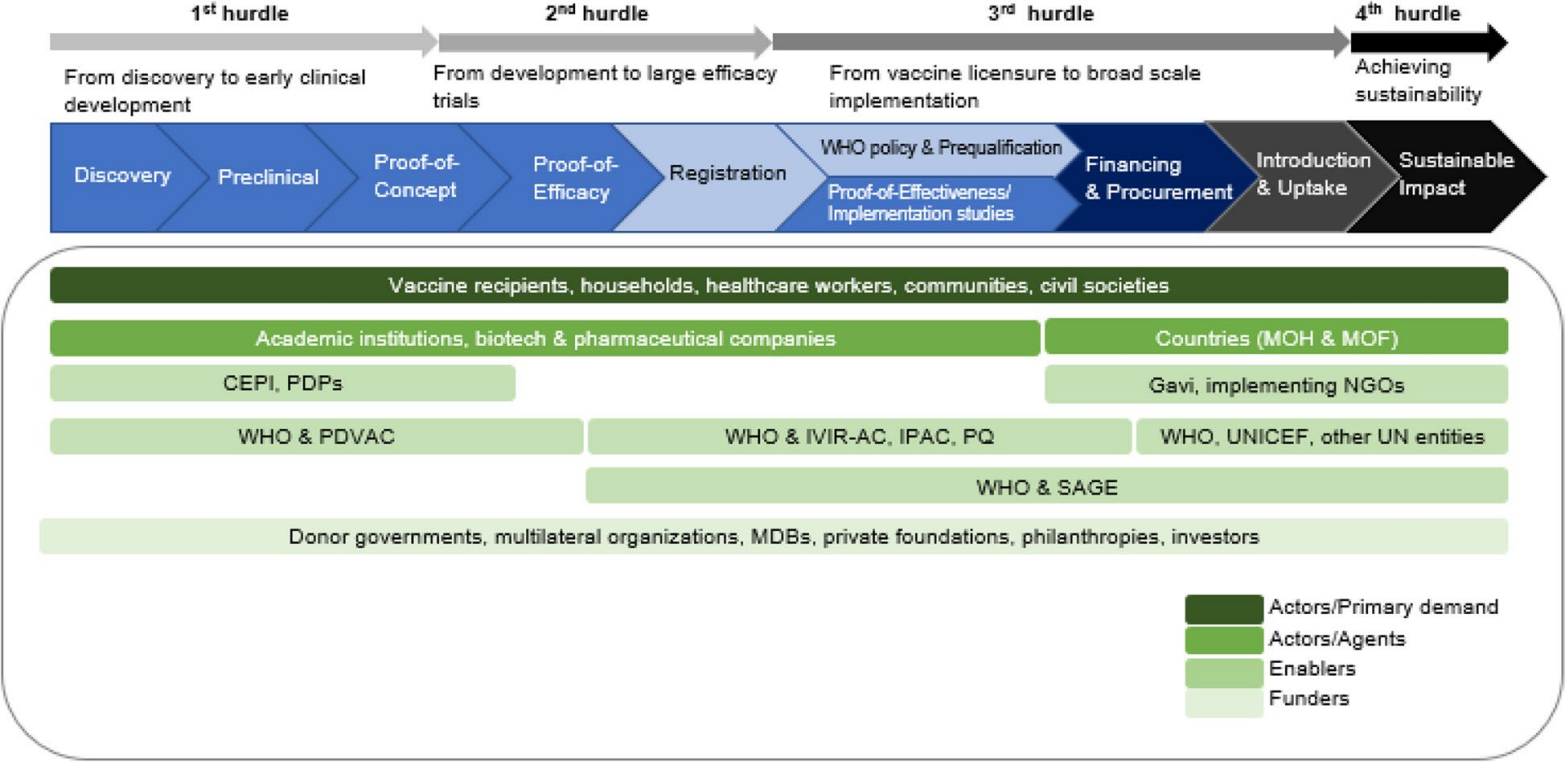
The “full value of vaccines” continuum: pathway, hurdles and stakeholders. MOH, Ministry of Health; MOF, Ministry of Finance; CEPI, Coalition for Epidemic Preparedness Innovations; PDPs, Product Development Partnerships; PDVAC, Product Development for Vaccines Advisory Committee; IVIR-AC, Immunization- and Vaccine-related Implementation Research Advisory Committee; IPAC, Immunization Practices Advisory Committee; PQ, prequalification; SAGE, Strategic Advisory Group of Experts on Immunization; MDB, Multilateral Development Bank

Piot et al. (2019) identify four major hurdles, or gaps, on the road to achieving population-level vaccine impact: first, one between discovery and early clinical development (specifically, proof-of-clinical concept), also known as the translation gap; second, one between early development and licensure, sometimes referred to as the second valley of death; third, one between licensure and subsequent introduction; and, fourth, one between scale-up of implementation and ensuring sustainable and equitable impact of immunization programmes (Fig. 1) [1]. While these hurdles represent seemingly distinct problems faced by different stakeholders along the vaccine value chain, they are in fact related and need to be addressed through an over-arching strategy integrated throughout vaccine development, licensure, policy-making, introduction and sustainable use.

The second valley of death is a particular challenge for vaccines for which the public health need is concentrated in low-income settings, because of uncertainties regarding demand and national uptake, which can discourage investment in-late stage of product development. An understanding of the factors affecting decision-making during late-stage product development, assessment by national regulatory authorities and introduction, including the availability of domestic financing, is often lacking.

Failure to consider and mitigate the aforementioned obstacles in a holistic way has hindered comprehensive progress and impact with vaccines, particularly in LMICs, but increasingly in other settings as well.

Recent examples include vaccines for hepatitis E virus, tuberculosis (TB), and group A *Streptococcus*, each of which was affected by one or more of the four major hurdles in the vaccine value chain. In the case of hepatitis E, two vaccines have completed Phase III trials and are licensed for use in China and India. However, the high cost of vaccine development and the limited market for hepatitis E vaccines have been major challenges to their wider availability [2].

Similarly, the development of new TB vaccines has faced significant challenges because of the complex disease course, the challenges of vaccine development and uncertain national demand, particularly for a vaccine that will not be deployed within the infant immunization programme. Several vaccine candidates are currently in clinical trials, but the investment in late-stage development is far from certain and the policy environment and implementation strategies are not well defined [3, 4].

For GAS a few vaccine candidates are in preclinical and early-stage clinical development, but more research is needed to identify optimal targets for protective immunity, particularly in low-income settings [5].

Coordination and communication across multiple stakeholders can be enhanced through mechanisms for the integration of diverse sets of information and the harmonization of divergent incentives. Efforts so far typically have been categorized in terms of ‘pull’ and ‘push’ mechanisms [6]. Financial ‘pull’ incentives include mechanisms such as advanced market commitments, market guarantees or prize systems. In addition, providing systematic information on demand preferences or desired product characteristics has also helped to incentivize manufacturers and reduce the uncertainties in the development of products suitable for use in public immunization programmes in LMICs.

Push mechanisms include financial incentives, such as grants, subsidies, co-financing arrangements, product-development partnerships, or in the form of technical assistance for country plan development, partner-coordination mechanisms, and social mobilization. These can help stakeholders to reduce risks and share costs, thereby facilitating the development and uptake of vaccines.

**Fig. 2:**
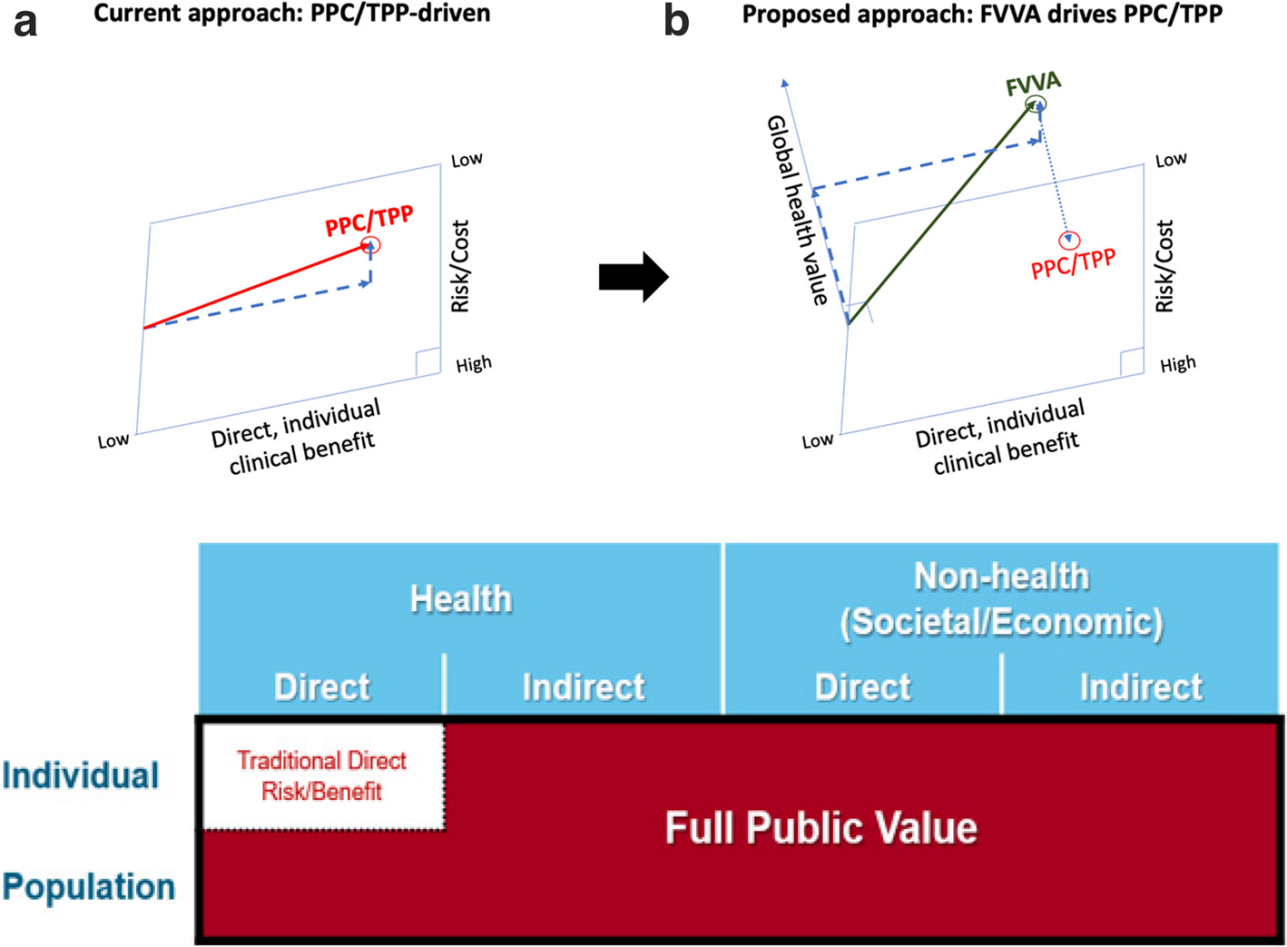
Current approach vs. proposed approach. **a** Traditional direct risk/benefit vs. full public value of vaccines. **b** Addition of global health value

While these kinds of mechanisms have improved coordination across stakeholders, there is a great diversity in approaches that countries take towards assessing the value of vaccines. WHO guidelines around the economic evaluation of vaccines advocate that such assessments should consider broad population-level effects of vaccines (such as herd protection, reduction of antimicrobial resistance (AMR) and serotype replacement), as well as the effects that vaccines can have on long-term human capital development (Fig. 2b) [7]. However, many countries only consider ‘narrow’ benefits such as gains in health, reduced health-care costs, and increased care-related productivity [8], although others increasingly assess and quantify the broader benefits of vaccination considering the wider effects such as herd protection, educational outcomes, equity, financial and programme synergies, impacts on public sector budgets, and macroeconomic consequences [9]. The broader approach implies a paradigm shift, with a wider range of benefits and costs integrated into discussions of commercial, regulatory and implementation policy [10]. However, to facilitate this quantitative assessment of broader benefits there is a need for greater standardization both between countries and between disease areas to facilitate international and cross-disease comparisons across different stakeholders.

Thus, adopting a comprehensive framework that both takes an end-to-end view of the vaccine development-to-uptake continuum (Fig. 1) and accounts for the broader benefits of vaccines has the potential to structure dialogue between stakeholders and foster coordination. An approach that provides a more holistic assessment is likely to address the major hurdles facing vaccine development and result in more sustainable and equitable vaccine development and introduction.

### Solution

We propose a new framework for considering the Full Value of Vaccine Assessments (FVVA) to guide the assessment and communication of the value of vaccines and to facilitate alignment among key stakeholders. As shown in Fig. 1, these stakeholders would include communities (e.g. potential. vaccine recipients, households), health care workers, vaccine agents (e.g., academic institutes, biotech and pharmaceutical companies), vaccine enablers (e.g. product-development partnerships (PDPs), the Coalition for Epidemic Preparedness Innovations (CEPI)) and funders (e.g. donor governments, international and multilateral organizations, multilateral development banks (MDBs)). Such a framework would inform decision-making related to investment in the development of vaccines intended for use in LMICs, as well as country-level decision-making related to introduction, especially in LMICs, by enabling greater cross-country and cross-disease comparability. The FVVA framework which builds on publications and events that took place over the last decade (see Supplement 1) introduces two concepts.

First, we argue that a number of adaptations are required in the individualist, welfarist methods of assessment that are still used in many countries to account for the evidence on broader impact. Second, we argue that the full value of vaccines derives not only from the results of assessments of benefits and costs (whether welfarist or broader in scope) but also rests on the robustness of the decision-making process itself.

The FVVA framework here aims to provide guiding principles that can promote country ownership of decision-making and priority setting. It is intended to address comprehensively, therefore, not only the hurdles hindering development, licensure, and introduction, but also those hindering equitable access and desired coverage required to achieve and sustain optimal population-level impact described in the Sustainable Development Goals (SDGs) and Immunization Agenda 2030 (IA2030). The framework will better integrate LMICs preferences and needs of LIMCs, while increasing the likelihood of achieving global goals for health and well-being. As a final preliminary remark, we demonstrate below that value propositions, investment cases, and business cases can all be seen as derivative use cases of FVVAs.

The FVVA framework incorporates two streams in Enlightenment ethics generally referred to as consequentialism and proceduralism that have dominated discussions around the assessment of health interventions, including immunization.

Consequentialism is “a philosophical view that actions should be judged by their outcomes”, through which actors can “capture and organize knowledge about states of the world in ways that are relevant to decision-making” [11]. This is the view that primarily motivates both what we have called the narrow and broader approaches to value assessment.

On the other hand, proceduralism is “a rules-based conception of decision-making according to which decisions are only partially (if at all) justified with reference to the goals stakeholders may have regarding outcomes, but where it is held that descriptive modelling (e.g. ‘deliberative discourse’) should structure and make explicit the reasons for actors’ preferred decisions, establishing the possibility of ‘accountability for reasonableness’” [11]. This view motivates an increased attention on the decision-making process itself.

These two views have typically been seen as mutually exclusive or even contradictory. However, modern commentators have argued that both are important components of just health policy and governance [12]. There is also a need to incorporate ethical systems that are not rooted mainly in Enlightenment thought, with its focus on the individual. This includes incorporating concerns from non-Western ethical systems such as communitarian values and relationships.

We assert that they can be harmonized and that best-practice health technology assessment (HTA) and other initiatives that we discuss below provide the broad outlines of how to incorporate both ethical views assessments of the value of vaccines. Specifically, in this paper we argue that (1) consequentialist frameworks should take a broader perspective and (2) that proceduralism must be integrated with consequentialist approaches, especially to ensure successful coordination and alignment of diverse stakeholders.

### Assessment: the need to adapt existing methods and tools

To estimate the broader benefits of vaccination, existing methods and tools need to be adapted to include additional types of benefit for which empirical evidence has become available in recent years, as well as novel concepts of value that incorporate risk, uncertainty, and/or equity (e.g. financial risk protection provided by vaccination [13]). Figure 2a is a simple graphical metaphor of the added dimension of FVVAs in relation to preferred product characteristics (PPCs) and target product profiles (TPPs), which tend to focus more on direct, individual-level benefits as well as risks and costs.

Our claim is that technologies cannot fully express characteristics that are valuable to stakeholders when this additional dimension (called here for illustration purposes “global health value”) is ignored or underestimated by stakeholders and decision-makers. Similarly, Fig. 2b demonstrates the narrower focus of TPPs, PPCs, economic evaluations, and HTA in relation to their potential scope. Appraisal of the evidence demonstrates that there is also a need for more experimental and observational studies to enhance the evidence base for the benefits of vaccination [9] broader than those included in existing conceptual frameworks [8, 14, 15]. Gessner et al. called for an inventory of evidence and an annual monitoring of progress on completeness [10].

There are a number of existing guidelines for assessment of value (Table 1). Based on the work of Lauer et al. (2020), the FVVA framework provides a set of organizing principles for the consideration and selection of assessment approaches [11]. Methodological and technical details belonging to the various approaches should be based on the recommendations of existing guidelines.

**Table 1:** Evidence-based assessment methods driven by policy questions and decision contexts

Assessment method	Policy question	Typical summary measure	Guidelines/examples
Effectiveness study	How effective is vaccine in improving health outcome?	• Efficacy/effectiveness outcomes • Health outcomes	• FDA GCP/Clinical Trial Guidance documents [16] • Reporting guidelines for main study types [17]
Vaccine impact modelling study	How effective is vaccine in improving health outcome?	• Children vaccinated/immunized • Cases/deaths averted • QALY gained/DALY averted • Bed days/outpatient visits averted	• ISPOR-SMDM Modelling Good Research Practices Task Force Report [18] • Review articles on mathematical models for epidemiology of infectious diseases [19, 20]
Costing study	What is the financial value of resources consumed to develop/introduce a new vaccine?	• Cost per dose; per infant; per fully immunized child	• Consensus statement on vaccine delivery cost [21] • How to cost immunization programmes: a practical guide on primary data collection and analysis (EPIC project) [22]
Cost of illness study (COI)	What is the economic burden that vaccine- preventable diseases imposes on the society?	• Total direct and indirect cost of a case due to a vaccine-preventable disease	• WHO manual for estimating the economic burden of seasonal influenza [23] • Methodological considerations for COI studies of enteric fever [24]
Cost-effectiveness analysis (CEA)/cost-utility analysis (CUA)	Is a vaccine or immunization programme economically justified according to the opportunity cost of health care spending?	• Incremental cost-effectiveness ratio (ICER) using natural units or QALYs, DALYs	• The iDSI Reference Case for Economic Evaluation [25] • Economic analysis of vaccination programmes: ISPOR Good practices for Outcomes Research Task Force report [26] • WHO guide to cost-effectiveness analysis [27, 28]
Extended cost-effectiveness analysis (ECEA)	With given cost, what is the effect of a vaccine on financial risk protection, health gains, and averted private expenditures across income quintiles?	• Financial protection afforded by expenditure, distributional aspects of outcomes	• Example: Extended Cost-Effectiveness Analysis for Health Policy Assessment: A Tutorial] [13]
Benefit–cost analysis	Is vaccine purchase economically justified according to individuals’ willingness to pay for health gains? What is the return on investment for a vaccine from the perspective of a vaccine manufacturer, funder or immunization programme?	• Benefit–cost ratio (BCR) • Positive net benefits • Return on investment (ROI), net present value (NPV), internal rate of investment (IRR)	• Reference Case Guidelines for Benefit–Cost Analysis in Global Health and Development [29]
Investment/business case case/value proposition	What type of evidence is needed to inform decisions or advocacy for development and introduction of vaccines as well as sustainable implementation of immunization programmes?	• Disease burden • Cost of investment • Impact of investment • Considerations for implementation	• Systematic review of existing examples [30]
Economic surplus analysis	How is global welfare in dollar terms generated by the uptake of vaccines distributed between vaccine producers vs. purchasers? Between HIC vs. LMICs? Across countries?	• Consumer surplus • Producer surplus • Total economic surplus	• Example: economic surplus analysis for HPV vaccines [31]
Budget impact analysis (BIA)	What will be the impact of adding a new vaccine on the budget of a stakeholder (i.e. annual budget of the Ministry of Health)?	• Difference in health system costs (with vs. without the intervention	• Budget Impact Analysis—Principles of Good Practice: Report of the ISPOR 2012 Budget Impact Analysis Good Practice II Taskforce [32]
Optimization modelling	Given a constrained budget, what is the best possible set of interventions including vaccine that maximizes/minimizes the target outcome?	• Minimization/maximization of outcome variable(s) of interest	• Economic analysis of vaccination programmes: an ISPOR Good Practices for Outcomes Research Task Force Report [26]
Fiscal impact modelling	What is the change in tax revenue and transfer payments attributable to changes in mortality and morbidity caused by a vaccine?	• Net present value (NPV) • Return on investment (ROI) • Benefit–cost ratio (BCR)	• Economic analysis of vaccination programmes: an ISPOR Good Practices for Outcomes Research Task Force Report [26]

In all economic evaluations, stakeholders and decision contexts are important since they drive the choice of inputs, outcomes and assessment methods. It is important to identify the policy, business question or decision context that is relevant to each stakeholder (Table 1). These factors drive the choice of assessment methods, by use of which outcomes of vaccination are compared against distinct types of costs (market-traded and non-market-traded inputs) borne by each stakeholder.

### Decision-making process: a focus on the agency of stakeholders

By considering both outcome (consequentialism) and the process of decision-making (proceduralism), the FVVA approach ensures that stakeholders involved are making decisions that are in the best interests of vaccine recipients while upholding ethical principles i.e. just and fair processes in decision-making.

Multi-criteria decision analysis (MCDA) is one example of a decision-making method that is both proceduralist and consequentialist [33]. Deliberative processes using either a qualitative or quantitative MCDA framework, can provide additional advantages, such as enhanced consensus building, identification of evidence gaps, and an increased likelihood of acceptance and implementation of decisions [34]. Global-level prioritization processes such as Gavi’s Vaccine Investment Strategy [35], the Vaccine Innovation Prioritization Strategy [36] and WHO’s Value Attribution Framework for Vaccines Against AMR [37], take a qualitative approach to MCDA and rely mainly on an evidence-informed deliberative process conducted by global decision-makers and domain-specific experts.

A deliberative approach is also a critical component of HTA processes, and forms part of the contextualization of evidence [38], based on transparent, accountable and evidence-informed political and social judgement at the local level [39]. The importance of participatory dialogue is also emphasized in the WHO ‘3Ds’ approach (data, dialogue and decision) to priority setting for universal health coverage [40].

The importance of incorporating a proceduralist approach was highlighted during the development of the WHO Country-led Assessment for Prioritization on Immunization (CAPACITI) project [41]. CAPACITI was initially based on total systems effectiveness (TSE) [42], a consequentialist framework aimed at optimizing the trade-offs between defined benefit and cost criteria through a quantitative MCDA model. For immunization programmes, the proceduralist approach allows the incorporation of national and local values and programme context to facilitate country-owned decision-making; for research and development, a deliberative process can allow LMICs to communicate a consensus view to product developers, strengthening the business case for investment. The country ownership of decision-making contributes to ensuring introduction, equitable vaccine access and coverage, and sustainable impact for Immunization Agenda 2030 [43].

### Communication: using FVVAs for value propositions, investment cases and business cases

Both consequentialist and proceduralist approaches adopt the perspective of specific stakeholders or decision-makers. An unaddressed question is: how do we facilitate dialogue *across* stakeholders focusing on different parts of the vaccine value chain be facilitated, to overcome hurdles to maximize the impact of vaccines?

There is a growing number of “value propositions”, “investment cases”, or “business cases” relating to vaccines and immunization programmes [44]. The Full Public Health Value Propositions for Vaccines (FPHVPs), the predecessor of the FVVA framework, were early to harmonize such work through the development of a comprehensive framework outlining the components of evidence needed to describe the full value of vaccines, with the goal of incentivizing vaccine development for LMIC markets. WHO’s Strategic Advisory Group of Experts on Immunization (SAGE) endorsed the approach in 2018, and the development of the approach was encouraged with strong consideration and representation of the end-user (country) perspective [45, 46]. Value propositions, investment cases, and business cases are all designed to facilitate the identification of the data needed for decision-making at each stage of development. Strategic alignment tools such as PPCs and TPPs specify the evidence required from clinical trials, implementation and market studies to guide funding and policy decisions as well as risk assessment [47]. They are intended to engage and align stakeholders more effectively along the continuum towards implementation.

A model that explicitly embeds stakeholders in the decision-making process, such as CAPACITI, will further enhance global dialogue. While the eventual creation of a feedback loop from end-users to R&D stakeholders is reflective of the ‘pull’ mechanism mentioned above, CAPACITI’s approach is intended to enable countries to articulate value propositions specific to local contexts and to communicate their needs in a more formalized, iterative process.

While the stated objectives of value propositions, investment cases, and business cases are typically to provide information for decision-making, they may have other objectives, including advocacy to convince a specific target audience to undertake a course of action (e.g. to invest in the development of vaccines). In practice, this advocacy function can become untethered from the requirements of scientific objectivity or even of the evidence considered. Assessments and decision aids intended to promote optimal outcomes both within and beyond the health sector should be by design independent of this advocacy function.

To mitigate the risk of loss of objectivity, the remit of a FVVA should always be aligned with the standard reference cases so as to avoid the appearance of “special pleading” for particular vaccines and to avoid explicit or hidden donor-driven agendas that are not aligned with country needs [48, 49]. While outcomes from FVVA-based approaches can be synthesized to support the development of value propositions, investment cases, and business cases for vaccines, the interpretation of the arguments and their application in a decision-making context will ultimately be at the discretion of the target audience. Comparing multiple arguments from different stakeholders can suggest insights on how incentives might be aligned and where and when mediation should happen, although such appraisals require strong technical capacity on the part of the decision-maker. The FVVA framework elucidates the aspects relevant to communication about the value of vaccines separately from the assessment of value and the priority-setting process so that the latter can be aligned with standard practices and broader global health goals, such as SDGs and IA2030, in support of achieving Universal Health Coverage.

### The Full of Value of Vaccines Assessment Framework and COVID-19 vaccines

**Fig. 3:**
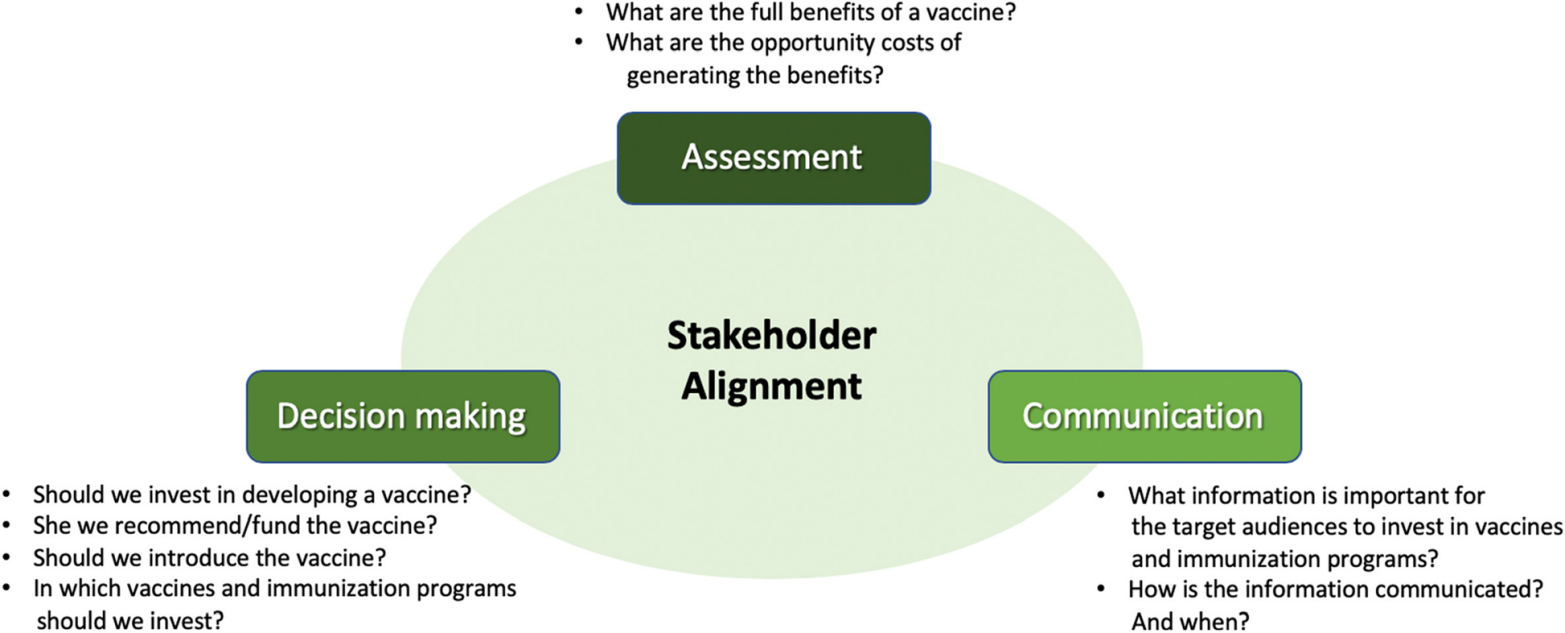
The full value of vaccines assessment (FVVA) framework: three functions

The implications of the FVVA concept for the development and implementation of vaccines for COVID-19 can be illustrated by addressing the extent to which (1) the broader assessment has been applied, (2) stakeholder dialogue has been considered, and (3) coordination across stakeholders has occurred (see Fig. 3).

#### Broader assessment of COVID-19 vaccines

Activities on the pathway to vaccine development, implementation, and impact are being de-risked by financial commitments and incentives and coordinated expeditiously by global stakeholders, through initiatives such as the COVAX facility as part of the Access to COVID-19 Tools (ACT) Accelerator [50]. There thus seemed to be a consensus on the part of virtually all stakeholders regarding the need, if not the means, to capture the full value of COVID-19 vaccine candidates, and the typical vaccine hurdles are therefore being effectively addressed. Considering investments as an economic impetus to get society “back to normal” [51] has resulted in an unprecedented number of candidates in development since the sequencing of the SARS-CoV-2 genome in the beginning of 2020 [52].

#### Stakeholder dialogue

Vaccines, like other health interventions, have their opponents as well as proponents, and decisions that might be made for the sake of an emergency response taken *purely on welfarist grounds* can have permanent implications if adverse consequences, real or perceived, lead to loss of confidence in vaccines or health decision-makers. This can clearly have durable long-term negative consequences for the full value of vaccines. Given the levels of resistance that have been witnessed to non-pharmaceutical interventions (NPIs) in some settings [53], active resistance to vaccines for COVID-19 seems likely regardless of the empirically derived risk–benefit profiles. As we argue here in the general case, likewise in the case of COVID-19 vaccines, it will also be *at least as important* to include the views of end-users and their representatives in a participatory decision-making process regarding implementation as well as tackling the scientific, manufacturing, regulatory, policy, financing, and logistical problems associated with immunization against COVID-19.

#### Coordination across stakeholders

After the COVID-19 vaccines received regulatory approval and policy recommendations for their widespread use, there remained the issue of how equitable access can be assured both between and within countries [54]. A technocratic approach to allocation needs to be integrated with participatory decision-making processes that incorporate technical, ethical, legal, regulatory, and policy perspectives to facilitate global dialogue on fair and equitable allocation of vaccines [55]. These processes should be based on both consequentialist and proceduralist principles and should be initiated concurrently with the ongoing development of COVID-19 vaccines and other new vaccines. Strategic alignment tools to assist include preferred product characteristics/target product profiles, strategic R&D roadmaps, integrated product development plans and preferred policy profiles/target policy profiles and WHO’s new Evidence Considerations for Vaccine Policy guidance [56]. All of these take into consideration the different perspectives of stakeholders, as well as the different decision-making criteria along the product development-policy-financing continuum.

Given the development and implementation of COVID-19 vaccines during the pandemic was widely discussed and highly relevant we demonstrate the value of the FVVA concept for COVID-19 vaccines in this paper. Although the COVID-19 vaccine development has been a monumental global effort that has yielded several vaccines in record time, the global distribution of these vaccines has been unequal, with LMICs experiencing significant disparities in access to the vaccines as many were not designed to be suitable for deployment in LMIC settings.

In future pandemics, vaccine development, financing, procurement and distribution need cooperation and coordination by global, regional and national stakeholders, government support, and investments in vaccine research and development capabilities in LMICs to ensure equitable access to vaccines using the FVVA concept as proposed in this paper. It could also be relevant for a new global public health security convention designed to optimize prevention, preparedness, and response to pandemic infectious diseases [57].

## Conclusions

The FVVA framework represents a set of coherent, organizing principles guiding the adoption of consequentialist tools while placing the autonomy and agency of stakeholders at the centre of decision-making. For the first comprehensive FVVA applications for “routine vaccines” studies, Lawn et al. (2020) [58], Gebreselasie et al. (2020) [59] and the recent WHO investment case for new TB vaccines [60] have demonstrated the need, usefulness and challenges of FVVA for maternal GBS and novel TB vaccines respectively, to different stakeholders for the further development of these vaccines by showing the potential impact of vaccine introduction in terms of health, economic, equity and financial risk protection in LMICs.

In summary, FVVAs facilitate the determination of the full value of vaccines and communication and coordination across stakeholders. Global-level efforts can be organized around the three core elements of the FVVA framework—assessment, stakeholder alignment and decision-making, and communication—to ensure that decision-making by national and other stakeholders is informed by the fullest possible understanding of the potential impact of a vaccine and is based on an appropriately inclusive process.

## Supplementary Information

**Additional file 1: Supplement 1.** Development of the FVVA. **Supplement 2.** Glossary of abbreviations and definitions.

## Abbreviations

AMR: Antimicrobial resistance
BCG: Bacillus Calmette-Guérin
BCR: Benefit-cost ratio
BIA: Budget impact analyses
CAPACITI: WHO Country-led Assessment for Prioritization on Immunization
CEA: Cost-effectiveness analyses
CEPI: Coalition for Epidemic Preparedness Innovations
COVID-19: Coronavirus disease for 2019
CUA: Cost-utility analyses
DALYs: Disability-adjusted life years
ECEA: Extended cost-effectiveness analyses
FPHVP: Full Public Health Value Propositions for Vaccines
FVVA: Full Value of Vaccines Assessments
GAS: Group A *Streptococcus*
Gavi: Global Vaccine Alliance
GBS: Group B *Streptococcus*
HICs: High-income countries
HTA: Health technology assessment
IA2030: Immunization Agenda 2030
ISPOR: The Professional Society for Health Economics and Outcomes Research
LMICs: Low- and middle-income countries
MDB: Multilateral development banks
NVP: Net present value
PDPs: Product-development partnerships
PPC: Preferred product characteristics
QALYs: Quality-adjusted life years
R&D: Research and development
ROI: Return on investment
SAGE: WHO’s Strategic Advisory Group of Experts on Immunization
SARS-CoV-2: Severe acute respiratory syndrome coronavirus 2
SDGs: Sustainable Development Goals
TB: Tuberculosis
TPP: Target product profiles
TSE: Total systems analyses
