# Supplementary material for: The Full Value of Vaccine Assessments (FVVA): a framework for assessing and communicating the value of vaccines for investment and introduction decision-making
Source: BMC Med. 2023 Jul 4;21:229. doi: 10.1186/s12916-023-02929-0 (PMC10318807; doi:10.1186/s12916-023-02929-0)
Supplement: Supplementary file 1 — Additional file 1: Supplement 1. Development of the FVVA. Supplement 2. Glossary of abbreviations and definitions. [file 12916_2023_2929_MOESM1_ESM.docx]

**Supplement 1: Development of the FVVA (could be a text box)**

The development of the FVVA framework has been inspired by seminal papers and events over the last decade.

- In their paper in 2008 Beutels and colleagues^1^ stated that vaccines need to be on a level playing field with other interventions. In particular features that are related to herd immunity, quality of life in young children, parental case and work loss, time preference, macroeconomics and tiered pricing relevant to decision making for vaccines, or for pharmaceuticals in general should be considered when assessing their cost-effectiveness.

- In early 2010s work on the broader economic impact of vaccines proposed a broader perspective in evaluations in different generic and vaccine preventable disease specific framework (e.g. by Barnighausen & Bloom^2,3^, reviews by Ozawa et al.^4^, Deogaonkar et al. ,Jit et al.^5,6^)

- Between 2015 and 2017 several meetings took place at the Fondation Mérieux place whereby Gessner and colleagues^7^ eventually proposed to go beyond the therapeutic paradigm applied to the evaluation of prophylactic vaccines that focuses on individual benefit-risk assessment. By contrast they propose a public health paradigm instead that considered population impact and community benefits against a range of outcomes. Follow up articles by David Kaslow and colleagues^8^ raised issues around problems in financing vaccine development relevant to LMICs.

- Subsequent statements from WHO’s advisory committees, including PDVAC, IVIR-AC and SAGE asking for clarity in this area (add refs).

- Funding of investment cases in GBS, GAS, Shigella, MR-MAP and TB^9^ which take a broader perspective.

- Gavi VIS criteria that have broader criteria: <https://www.gavi.org/our-alliance/strategy/vaccine-investment-strateg>

- WHO AMR framework leveraging Vaccines to Reduce Antibiotic Use and Prevent Antimicrobial Resistance: An Action Framework:

<https://cdn.who.int/media/docs/default-source/immunization/product-and-delivery-research/action-framework-final.pdf?sfvrsn=13c119f3_5&download=true>

- Possibly we could also mention the relevance to COVID-19 vaccines where a pure health sector perspective alone is clearly too narrow to capture all the benefits that society is interested in, and where WHO SAGE has explicitly laid down ethical principles for vaccine prioritisation.

- In preparation of the IA2030 global strategy the full benefits of vaccines to everyone contributing to good health and well-being play a prominent role.

**Supplement 2: Glossary of abbreviations and definitions**

2.1 Abbreviations

CAPACITI: Country-led Assessment for Prioritization of Immunisation

CEPI: The Coalition for Epidemic Preparedness Innovations

FPVHP: Full Public Health Value Propositions for Vaccines

FVVA: Full Value of Vaccines Assessment

HTA: Health Technology Assessment

IVIR-AC: Immunization- and Vaccine-related Implementation Research Advisory Committee

IPAC: Immunization Practices Advisory Committee

PDP: Product Development Partnerships (PDPs)

PDVAC: Product Development for Vaccines Advisory Committee

PPC: Preferred Product Characteristics

SAGE: Strategic Advisory Group of Experts on Immunization

TPP: Target Product Profiles

TSE: Total Systems Effectiveness

MDB: Multilateral Development Bank

2.2 Definitions

| Terms | Definitions |
| --- | --- |
| Preferred Product Characteristics (PPC) | PPCs are developed by WHO IVB and provide guidance as to WHO’s preferences for new vaccines in priority disease areas. The objective is to promote the development of vaccines with optimal effectiveness and suitability, for use in LMICs, thereby maximizing global vaccine impact. |
| Target Product Profiles (TPP) | The TPPs are pathogen rather than product specific and define a mandatory set of product attribute. The intent of WHO TPPs is to provide early technical guidance into the various product-specific vaccine TPPs that are developed by individual vaccine manufacturers |
| Health Technology Assessment (HTA) | A multidisciplinary process that uses explicit methods to determine the value of a health technology at different points in its lifecycle. The purpose is to inform decision-making in order to promote an equitable, efficient, and high-quality health system |
| Investment cases, business cases, value propositions | A body of work, as a compilation of outcomes from existing analyses and studies, that aims to provide information needed for decisions around technical or financial support for vaccine introduction by donors and country stakeholders, or investment decisions on vaccine development made by donors or private investors. Some aim to advocate for specific goals or agenda related to disease control and immunization programs. |
| Full Value of Vaccines Assessment (FVVA) | A framework that guides assessment and communication of the value of vaccines and informs decision making around vaccine development and introduction as well as sustainable implementation of immunization programs. |

1. Beutels P, Scuffham PA, MacIntyre CR. Funding of drugs: do vaccines warrant a different approach? *Lancet Infect Dis*. 2008;8(11):727-733. doi:10.1016/S1473-3099(08)70258-5

2. Bärnighausen T, Bloom DE, Canning D, et al. Rethinking the benefits and costs of childhood vaccination: The example of the Haemophilus influenzae type b vaccine. *Vaccine*. 2011;29(13):2371-2380. doi:https://doi.org/10.1016/j.vaccine.2010.11.090

3. Bärnighausen T, Berkley S, Bhutta ZA, et al. Reassessing the value of vaccines. *Lancet Glob Heal*. 2014;2(5). doi:10.1016/S2214-109X(13)70170-0

4. Ozawa S, Mirelman A, Stack ML, Walker DG, Levine OS. Cost-effectiveness and economic benefits of vaccines in low- and middle-income countries: A systematic review. *Vaccine*. 2012;31(1):96-108. doi:https://doi.org/10.1016/j.vaccine.2012.10.103

5. Jit M, Hutubessy R, Png ME, et al. The broader economic impact of vaccination: reviewing and appraising the strength of evidence. *BMC Med*. 2015;13(1):209. doi:10.1186/s12916-015-0446-9

6. Deogaonkar R, Hutubessy R, Van Der Putten I, Evers S, Jit M. Systematic review of studies evaluating the broader economic impact of vaccination in low and middle income countries. *BMC Public Health*. 2012;12(1). doi:10.1186/1471-2458-12-878

7. Gessner BD, Kaslow D, Louis J, et al. Estimating the full public health value of vaccination. *Vaccine*. 2017;35(46):6255-6263. doi:https://doi.org/10.1016/j.vaccine.2017.09.048

8. Kaslow DC, Black S, Bloom DE, Datla M, Salisbury D, Rappuoli R. Vaccine candidates for poor nations are going to waste. *Nature*. 2018;564(7736):337-339. doi:10.1038/d41586-018-07758-3

9. Gebreselassie N, Hutubessy R, Vekemans J, den Boon S, Kasaeva T, Zignol M. The case for assessing the full value of new tuberculosis vaccines. *Eur Respir J*. 2020;55(3). doi:10.1183/13993003.02414-2019

10. Lauer, JA; Morton, A; Culyer, AJ; Chalkidou K. *What Counts in Economic Evaluations in Health? Benefit-Cost Analysis Compared to Other Forms of Economic Evaluations. (Health Systems Financing and Governance, Working Paper No. 14).* Geneva; 2020.

11. Lauer A.J, Morton, A, Culyer A, Chalkidou K. *What Counts in Economic Evaluations in Health? Benefit-Cost Analysis Compared to Other Forms of Economic Evaluations.*; 2019.

12. Ruger JP. Global health justice and governance. *Am J Bioeth*. 2012;12(12):35-54. doi:10.1080/15265161.2012.733060
